# Supplementary material for: A short scale for measuring attitudes towards the doctor-patient relationship: psychometric properties and measurement invariance of the German Patient-Practitioner-Orientation Scale (PPOS-D6)
Source: PeerJ. 2021 Dec 8;9:e12604. doi: 10.7717/peerj.12604 (PMC8667738; doi:10.7717/peerj.12604)
Supplement: Supplemental Information 1 — *An English translation is added for understanding purposes only. An evaluation of the psychometric properties of the English items was not part of this study. [file peerj-09-12604-s001.docx]

| **Appendix 1. PPOS-D6 Items in German and English** | | | |
| --- | --- | --- | --- |
| Factor | No. | Item German | Item English* |
| Sharing | 2 | Patienten sollten sich auf das Wissen ihrer Ärzte verlassen und nicht versuchen, sich selber über ihre Erkrankung zu informieren. | Patients should rely on the knowledge of their doctors and not try to inform themselves about their condition. |
|  | 5 | Wenn Patienten eine andere Meinung haben als ihre Ärzte, zeigt das, dass sie ihre Ärzte nicht respektieren und ihnen nicht vertrauen. | When patients have a different opinion than their doctors, it shows that they do not respect and trust their doctors. |
|  | 6 | Der Patient muss sich immer darüber im Klaren sein, dass der Arzt die Verantwortung trägt. | The patient must always be aware that the doctor is in charge. |
| Caring | 1 | Der wichtigste Teil der ärztlichen Visite ist die körperliche Untersuchung. | The most important part of the medical visit is the physical examination. |
|  | 3 | Wenn Ärzte viele Fragen zur persönlichen Situation eines Patienten stellen, mischen sie sich zu sehr in private Angelegenheiten ein. | When doctors ask many questions about a patient's personal situation, they interfere too much in private matters. |
|  | 4 | Wenn Ärzte wirklich gut sind in Diagnostik und Therapie, ist ihr Umgang mit den Patienten nicht so wichtig. | If doctors are really good at diagnostics and therapy, their interaction with patients is not as important. |

*An English translation is added for understanding purposes only. An evaluation of the psychometric properties of the English items was not part of this study.
