# Supplementary material for: A short scale for measuring attitudes towards the doctor-patient relationship: psychometric properties and measurement invariance of the German Patient-Practitioner-Orientation Scale (PPOS-D6)
Source: PeerJ. 2021 Dec 8;9:e12604. doi: 10.7717/peerj.12604 (PMC8667738; doi:10.7717/peerj.12604)
Supplement: Supplemental Information 2 — Note. ** p < .01, * p < .05. [file peerj-09-12604-s002.docx]

| **Appendix 2. Mean values, standard deviation, skewness, kurtosis and zero-order correlations of manifest items (N = 290)** | | | | | | | |
| --- | --- | --- | --- | --- | --- | --- | --- |
|  | Variable | 1 | 2 | 3 | 4 | 5 | 6 |
| 1 | Item 1 | 1 |  |  |  |  |  |
| 2 | Item 2 | .21^**^ | 1 |  |  |  |  |
| 3 | Item 3 | 0.10 | 0.07 | 1 |  |  |  |
| 4 | Item 4 | .17^**^ | 0.08 | .14^*^ | 1 |  |  |
| 5 | Item 5 | 0.10 | .27^**^ | 0.11 | .21^**^ | 1 |  |
| 6 | Item 6 | 0.10 | .27^**^ | 0.04 | 0.06 | .22^**^ | 1 |
|  | Mean | 2.89 | 3.55 | 4.71 | 5.38 | 4.56 | 3.04 |
|  | Standard deviation | 1.03 | 1.36 | 0.97 | 0.78 | 1.08 | 1.26 |
|  | Skewness | 0.55 | 0.06 | -0.99 | -1.26 | -0.51 | 0.32 |
|  | Kurtosis | 0.52 | -0.73 | 1.80 | 1.73 | -0.03 | -0.33 |

Note. ** p < .01, * p < .05.
